# Why using bed nets is a challenge among minority populations in Central Vietnam

**DOI:** 10.1186/s12936-022-04114-9

**Published:** 2022-03-15

**Authors:** Thuan Thi Nguyen, Xa Xuan Nguyen, Marta Wilson-Barthes, Ikumi Sawada, Joan Muela, Susanna Hausmann-Muela, Thanh Vinh Pham, Hong Van Nguyen, Van Van Nguyen, Duong Thanh Tran, Charlotte Gryseels, Umberto D’Alessandro, Koen Peeters Grietens, Annette Erhart

**Affiliations:** 1 Socio-Ecological Health Research Unit, Department of Public Health, Institute of Tropical Medicine, Antwerp, Belgium; 2 National Institute of Malariology, Parasitology and Entomology, Hanoi, Vietnam; 3 International Health Institute, Brown University School of Public Health, Providence, USA; 4 Department of Clinical Tropical Medicine, Institute of Tropical Medicine, Graduate School of Biomedical Science, Nagasaki University, Nagasaki, Japan; 5 University Ramon I Virgili, Tarragona, Spain; 6 Partners for Applied Social Sciences, PASS International, Tessenderlo, Belgium; 7 Center for Disease Control, Quang Nam Province, Vietnam; 8 School of Tropical Medicine and Global Health, Nagasaki University, Nagasaki, Japan; 9 Medical Research Council Unit The Gambia at the London School of Hygiene and Tropical Medicine, Fajara, The Gambia

**Keywords:** Vietnam, Forest malaria, Ethnic minorities, Insecticide-treated nets, Bed net use, Social determinants, Vector control strategies, Mixed-methods study

## Abstract

**Background:**

Despite freely distributed insecticide-treated nets (ITNs) and health information campaigns to increase their use among populations at risk, malaria transmission persists in forested areas in Vietnam, especially among ethnic minority communities. A mixed-methods study was conducted in four villages of Ca Dong and M’nong ethnicity in Central Vietnam between 2009 and 2011 to assess factors limiting the uptake of ITNs.

**Methods:**

The mixed-methods research design consisted of a qualitative study to explore the context and barriers to ITN use, and a cross-sectional household survey (n = 141) to quantify factors for limited and appropriate net use.

**Results:**

The Ca Dong and M’nong’s livelihood was dependent on swidden farming in the forest. Poverty-related factors, including the lack of beds, blankets, the practice of sleeping around the kitchen fire and deteriorated ITNs due to open housing structures, were reasons for alternative and non-use of ITNs. When household members stayed overnight in plot huts at fields, ITNs were even more unavailable and easily deteriorated. 72.5% of households reported having received one net for every two persons, and 82.2% of participants reported to have used ITNs the night before the survey. However, only 18.4% of participants were estimated to be effectively protected by ITNs after accounting for the availability of torn ITNs and the way ITNs were used, for example as blankets, at both village and fields. Multi-variable logistic regression showed the effect of four significant factors for appropriate ITN use: i) being female (AOR = 8.08; p = 0.009); ii) aware of mosquito bites as the sole cause of malaria (AOR = 7.43; p = 0.008); iii) not sleeping around the kitchen fire (AOR = 24.57; p = 0.001); and iv) having sufficient number of ITNs in the household (AOR = 21.69; p = 0.001).

**Conclusion:**

This study showed how social factors rooted in poverty and swidden agriculture limited the effective use of ITNs, despite high coverage, among ethnic minority populations in Central Vietnam. An in-depth understanding of the local context is essential to develop specific indicators for measuring ITN use.

## Background

Malaria control efforts in Vietnam contributed to a sharp decline of the disease burden with a decrease of 97% in morbidity and 99.8% in mortality between 1992 and 2014 [1]. In the past ten years, malaria transmission has been interrupted in most of the northern and southern provinces, however, the remaining challenge to malaria elimination is residual transmission in south-central Vietnam where several thousand cases are still reported annually [2–4]. Since 1992, and following recommendations of the World Health Organization (WHO), the National Malaria Control Programme (NMCP) has provided free-of-charge early detection and treatment of malaria together with integrated vector control interventions, including indoor residual spraying (IRS) and insecticide-treated nets (ITNs) to the population living in endemic areas [5–7]. Between 2003 and 2019, the estimated population at risk of malaria (*i.e.* living in malaria endemic areas) decreased from 23.3% and 6.5% [4, 8] of the Vietnamese population.

Universal coverage of ITNs and long-lasting insecticidal nets (LLINs) for populations at risk of malaria has been repeatedly proven as a highly cost-effective public health intervention to avert malaria infections and deaths [9–12]. From 2010, Vietnam adopted a national strategy aiming for malaria elimination that included the distribution of LLINs, ITNs, and IRS, in addition to health information-education-communication (IEC) to boost the uptake of these measures by populations in malaria-endemic areas. This strategy was funded by both national and international sources with fluctuating annual funding [8, 13, 14]. As a result of this unstable funding, the coverage of ITNs and LLINs—defined as two persons per double bed net—distributed to all households living in high transmission areas (following the official census), fluctuated between 30% in the early 2000s and 100% in 2010 [2, 14]. Despite financial challenges, Vietnam continues to aim for universal coverage of LLINs as one of the main interventions for malaria elimination by 2030.

High bed net coverage, however, does not necessarily equate with effective use, and the sub-optimal use of freely distributed bed nets has been reported in different settings, particularly among ethnic minority populations [15, 16]. In Vietnam, the main risk groups for malaria are ethnic minorities and migrant workers whose varied demographic characteristics, forest work and mobility have been a challenge for standardized vector control interventions [17–22]. This paper reports the results of a mixed-methods study assessing factors limiting the uptake of ITNs in an ethnic minority setting in a remote forested area of Central Vietnam. This study was embedded in a larger community-based cohort study on the epidemiology of *Plasmodium vivax* [23, 24].

## Methods

### Study site and population

The study was conducted in four villages of Tra Leng and Tra Don communes in Nam Tra My district of Quang Nam province. In the 2009 census, the total population consisted of 1810 individuals (352 households) with a majority being of M’nong and Ca Dong ethnicities and a minority of Kinh, the dominant ethnic group in Vietnam [23]. Despite state-led socio-economic interventions among ethnic minorities, according to the 2010 national survey on household living standards, the two ethnic groups were among those presenting the highest poverty rates [25].

Malaria transmission was perennial in the study area with two peaks in May–June and October–November. In April 2009, malaria prevalence was determined at 7.8% with a majority of *Plasmodium falciparum* infections (81.4%) being confirmed by light microscopy. However, by molecular techniques (PCR), the prevalence was three times higher (22.6%) with *P. vivax* accounting for a higher proportion of cases (43.2%) [23]. The main vector responsible for local malaria transmission was *Anopheles dirus*, which is highly anthropophilic and displays behavioural complexity, including outdoor biting and early feeding [26]. Exposure to infected mosquitoes has been shown to increase significantly when people stay in the forest at night, including staying at fields [27].

### Study design

The study used a two-stage exploratory mixed-methods design, starting with a qualitative strand followed by a quantitative strand [28]. In the qualitative strand, the researchers used an ethnographic approach to data collection to gain an in-depth understanding of the local context and barriers to effective bed net use. Preliminary findings from the qualitative strand were used to inform the design of a structured questionnaire in the quantitative strand, consisting of a household survey to complement and triangulate previous findings [29].

### Qualitative strand

#### Data collection

Qualitative data were collected in the four study villages during three periods of fieldwork between April 2009 and January 2011. The researchers stayed in the villages to get familiarized with local people. Data were collected in both rainy and dry seasons through participant observation (PO), informal conversations (ICs), in-depth interviews (IDIs) and informal group discussions (IGDs). For PO, the researchers immersed themselves in the study context by participating in everyday activities in the communities, observing events, and acquiring an in-depth understanding of the local social and cultural aspects that were unspoken or taken for granted. During PO, the researchers engaged with local people in informal conversations to build up rapport and trust. ICs were often spontaneous and occurred as part of the social interaction between the researchers and local people. As part of the fieldwork, the researchers took notes during the POs and ICs, and completed their notes immediately after each PO or IC. Once trust was built with the participants and response bias due to social desirability was minimized, the researchers proceeded to IDIs and IGDs to discuss aspects of bed net use with participants. IDIs and IGDs were conducted in Vietnamese and local languages with the help of translators when needed. Informal rather than formal interviews and discussions were preferred as formality exacerbated social hierarchies among Kinh, M’nong, and Ca Dong participants, and was expected to increase socially desirable responses. IDIs and IGDs were audio-recorded, transcribed in Vietnamese, and translated into English.

#### Sampling

The sampling strategy was theoretical [30]. During fieldwork, the researcher gradually included people with different profiles and experiences for maximum variation. The selection of participants was based on residence, experience in health services, ethnicity, occupation, gender, the use of bed nets and understanding of malaria. Included in the sample were men, women, young adults, and elderly people with diverse profiles, including farmers, malaria patients, school children, teachers, health professionals and representatives of the local authorities.

#### Data analysis

The researchers followed a continuous, flexible and iterative process for data analysis. During fieldwork, emergent codes and themes were discussed among the research team until consensus was obtained. Preliminary data were intermittently analysed to adapt interview guides. Emerging themes were continuously identified and new research hypotheses were further tested until no new findings emerged (*i.e.* data saturation) [31]. The researchers used a retroductive approach to data analysis which combines both inductive (emerging results) and deductive (existing theory) analysis [32]. Once data collection was completed, additional data coding was carried out. The researchers stored and analysed data using NVivo 10 Qualitative Analysis software (QSR International Pry Ltd. Cardigan UK).

## Quantitative strand

### Data collection

Quantitative data were collected from October to December 2010. Preliminary results from the qualitative strand on kinship, resettlement patterns, mobility, sleeping places, forest activities, bed net use, and health-seeking behaviour were used to inform the design of the quantitative survey. A paper-based questionnaire (in English and Vietnamese), consisting of structured questions for the household-level and the individual-level, was developed and field-tested before finalization in Vietnamese. The household-level questions were asked to household leaders (HHLs) to quantify net ownership, mobility patterns, and sleeping places. The individual-level questions were asked to individual household members (HHMs) to quantify their knowledge of malaria causation as promoted by ITN distribution campaigns, exposure to the vector and bed net use. Parents or guardians answered on behalf of minors. The survey questionnaire was administered by experienced field workers who had gained the confidence of the study populations owing to their previous research in the study setting. Prior to the survey, field workers were trained to administer the questionnaire to the study population. During the survey, field workers validated self-reporting bed net ownership and status through direct observation. No systematic measurement or counting of holes and tears was used to assess bed net status. Nets were considered torn when respondents stated they were broken and that mosquitoes entered the nets.

### Sampling

Village 1, where the highest number of vivax malaria cases was reported in the main cohort study in 2009, was selected for the survey [24]. The sampling frame consisted of households registered in the 2009 census, who were all invited to answer the questionnaire. HHLs were those identified by the census, while HHMs were family members, consisting of both adults and children, who self-reported that they had had malaria prior to the survey. Adults were defined as participants who were 16 years old or older. Field workers visited households with absent members up to four times to reduce the non-participation rate.

### Data analysis

Data were entered using Epi-Info 6.04 and analysed using Stata 12.0 (Stata Corporation, College Station, Texas, USA). Summary statistics were done using Chi-square- or Fisher’s exact tests accordingly for differences between categorical variables, while Student’s t-test or Wilcoxon rank-sum tests were used to compare continuous variables as required (significant p < 0.05). The survey design was taken into account using the “svy” command in Stata with household as the primary sampling unit (*i.e*., “svyset idhouse”; no other parameter used). A multi-variable analysis using survey logistic regression was conducted to determine the effect of independent risk factors (such as gender, education, occupation, knowledge of malaria causation, sufficient coverage of nets and blankets, and sleeping around the kitchen fire) on the appropriate net use. The latter was defined as reported sleeping under a hung-up net the night before the survey. The final model was built by selecting all variables that were significantly associated with the outcome (*i.e.* appropriate use of net) in the univariate analysis (significant p-value = 0.1) and using a backward stepwise selection method at a 10% significance (p < 0.1).

The socio-economic status (SES) of households was categorized as lowest, low, and higher SES according to their livestock ownership as defined by the larger study [23]. Bed net status was classified as “broken”- when a net had tears or holes allowing insects to enter, and “intact”- when a net had no tears or holes. Sufficient bed net ownership was defined as one net for every two persons (based on the number of observed nets in the households by field workers), while knowledge of malaria causation was the calculation of participants who acknowledged mosquito bites as the sole causation of malaria. Actual bed net use is usually very difficult to measure as it does not equate to reported bed net use [15, 33]. Therefore, a group of indicators was considered for the analysis including frequency of net use, sleeping places, multiple residences (*i.e.* village house and plot hut), net ownership at the plot huts, and whether the net was hung up at both village house and plot hut. A composite variable for bed net use was generated as follows: (i) “effectively protected by ITNs” for participants who reported they “always” slept under a hung-up net at both their village house and plot hut; or, reported doing so at the village and did not have a plot hut; (ii) “unprotected by ITNs” for participants who reported that they inconsistently slept under a net at their village house, and/or at fields.

### Ethical considerations

This study was approved by the Ethical Committee of the National Institute of Malariology, Parasitology and Entomology (NIMPE) in Hanoi, Vietnam (approval decision 362/QHQT), and the Institutional Review Board of the Institute of Tropical Medicine in Antwerp, Belgium (IRB/AB/dvm/183). All fieldwork in the qualitative strand followed the Code of Ethics of the American Anthropological Association (AAA) [34]. Village leaders from both ethnic groups and representatives of the Commune People’s Committee provided their consensus after having been informed on the study objectives and procedures. The use of oral informed consent was applied in the qualitative strand in which participants agreed to take part in the study after receiving explanation by the researchers about the study objectives, and the participant’s rights to answer or not any questions as well as to end the interview or discussion at any time as the participant wished to. The choice of oral consent was made in the consideration of the low literacy among the study population, and the sensitiveness of the topic, *e.g.* people’s sleeping places and their feedback on the nets given by the government. The researchers treated qualitative data with the highest confidentiality and stored data in password-protected devices. Participants in the survey were asked to consent before answering the questionnaire. When participants were under 16 years old, their parents or guardians were asked to consent and respond on behalf of the minors. All participants were informed of the intended use of the study results for scientific publication, and their responses were anonymized as to protect their access to and use of public health services.

## Results

### Study participants

The qualitative strand included 20 IDIs, 23 ICs, three group discussions and POs conducted by the researchers during 3 months. Participants were men and women in different age groups and had different professions such as farmers, plantation workers, students, teachers, health workers, local authorities and leaders. 141 individuals participated in the quantitative strand, including 80 HHLs and 61 HHMs. Of 61 HHMs, there were 15 adults and 46 children. The number of participating households accounted for 98.8% (80/81) of all households living in village 1. Demographic characteristics of survey participants are presented in Table 1. The M’nong and Ca Dong practiced swidden agriculture; both groups were poor and lived in remote locations.

**Table 1:** Reported ITN use and sleeping patterns amongst study participants

Individual survey (N = 141)	n	%	95% CI
Gender			
Female	74	52.5	[44.1; 60.8]
Male	67	47.5	[39.2; 55.9]
Age			
Median age of adults, range, IQR (N = 80)	27, (16; 88), IQR: 22–38		
Median age of children, range, IQR (N = 61)	7 (1; 15), IQR: 3–10		
Ethnicity			
Ca Dong	9	6.4	[2.3; 10.5]
M’nong	132	93.6	[89.5; 97.7]
Education (N = 117, excluding children under 6)			
Not having attended any school	18	15.4	[8.7; 22.1]
Attended primary school	49	41.9	[32.8; 50.9]
Attended secondary school	34	29.0	[20.7; 37.1]
Attended high school	7	6.0	[1.6; 10.3]
No answer	9	7.7	[2.7; 12.5]
Reported frequency of sleeping under a hung-up net (N = 141)			
Always	109	77.3	[69.7; 83.4]
Sometime	12	8.5	[4.9; 14.3]
Never	20	14.2	[9.4; 20.9]
Reported reasons to sleep under a hung-up net (N = 121)			
Warmth	2	1.6	[0.3; 7.9]
Protection from malaria	73	60.3	[47.0; 69.7]
Nuisance of mosquitoes/small insects	9	7.5	[1.5; 12.2]
Protection from mosquitoes, but I don't know why	15	12.4	[7.1; 23.2]
Fever	7	5.8	[2.3; 14.2]
Other/No answer	15	12.4	[7.7; 19.4]
Reported ITN use the night before the survey (N = 141)			
Yes	116	82.2	[75.8; 24.1]
No	25	17.7	[11.3; 24.1]
If yes, reported using the ITN as a blanket or a hung-up net? (N = 116)			
As a hung-up net	9	7.8	[2.8; 9.7]
As a blanket	107	92.2	[87.3; 97.1]

Household survey (N = 80)	n	%	95% CI
Median number of people in the household	Range, IQR 5 (1; 10); IQR: 4–6		
Median number of people per net	Range, IQR 2; (0; 5); IQR: 2–3		
Observed ownership of ITNs in households (N = 80)			
No answer	2	2.5	
1–2persons/net	58	72.5	[62.5; 82.5]
≥ 3 persons/net	20	25	[15.3; 34.7]
Observed status of ITNs in households (N = 78)			
Intact nets	19	24.3	[14.61 34.1]
Less than 50%	8	10.2	[3.45; 17.14]
More than 50% (< 100%)	25	32.0	[21.6; 42.6]
100% nets were broken	26	33.3	[22.6; 44.0]
Reported frequency of ITN washing (N = 78)			
Every week	28	35.9	[26.1; 47.0]
Once every 2 weeks	17	21.8	[14.1; 32.1]
Once every 3–4 weeks	24	30.8	[21.6; 41.7]
Less than once a month	9	11.5	[6.2; 20.5]
Reported duration until the ITN was broken (N = 78)			
< 6 months	1	1.3	[0.2; 6.9]
7–12 months	13	16.7	[1.0; 26.4]
13–24 months	29	37.2	[27.3; 48.3]
≥ 25 months	35	44.9	[34.3; 55.9]
Reported alternative use of broken ITNs (N = 59)			
Baby carrier	6	10.2	[3.8; 20.8]
Scrubbing the floor	15	25.4	[14.9; 38.4]
Blanket	20	33.9	[22.0; 47.3]
Pillow	29	49.2	[35.8; 62.5]
Fishing net	17	28.8	[17.7; 42.0]
Participants who reported to sleep around the kitchen fire (N = 80)			
Yes	48	60.0	[49.02; 71.0]
No	32	40.0	[29.02; 51.0]
Participants who reported that smoke in the kitchen drove mosquitoes away (N = 80)			
Yes	60	75.0	[65.3; 84.7]
No	19	23.8	[14.2; 33.3]
No answer	1	1.3	

### ITN/LLIN coverage and use

Prior to the study, the local population had received ITNs from the NMCP in 1998 and 2004. Participants said that between 2009 and 2010, they received LLINs for double beds from the government. At the time of the survey, households had both ITNs and LLINs (hereafter referred to as “ITNs”) at home. Participants perceived ITNs as part of the state’s subsidies to poor ethnic minority populations. They were hesitant to share their critical feedback on ITNs due to fears of being excluded from future free-of-charge public services, including health services if their information was disclosed to outsiders and local authorities. Concerning the distribution of ITNs, some participants said they received fewer ITNs than the recommended quantity, one net for every two adults, as advised by the NMCP. They said that while people did not object to using ITNs, they felt that these nets did not offer their preferred features. Commercial nets had characteristics that people preferred such as different colours, decorative contours, and an opening to enter the net (which ITNs did not have). However, the lack of financial means was the main reason for many not to buy nets in addition to the freely distributed LLINs. Field workers observed, at the time of the survey, that 72.5% (59/80) of households had at least one ITN for a maximum of two people. In 24.3% (19/78) of households, ITNs were observed to be still intact while in another 33.3% (26/78) of households they were broken (Table 1), leaving the rest of households (42.2%, 33/78) with various amounts of both intact and broken nets.

**Fig. 1:**
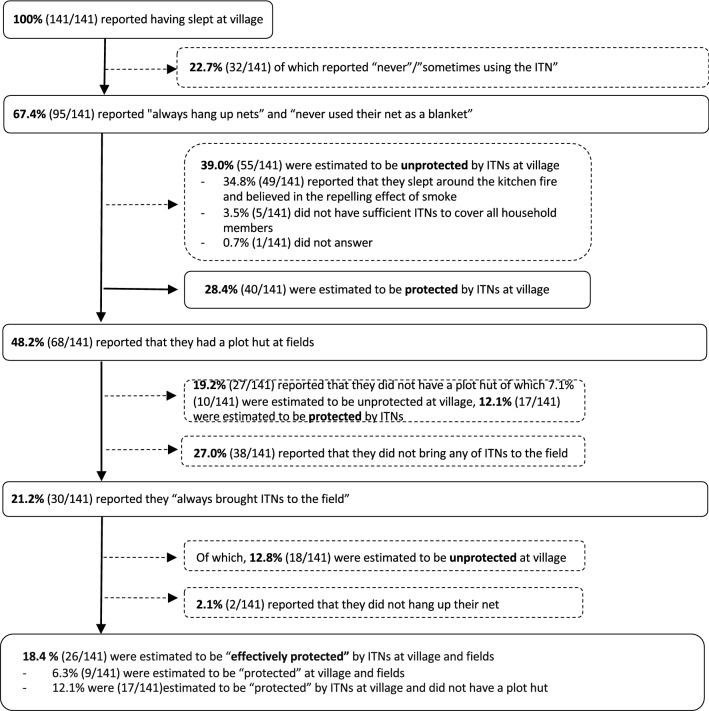
Estimate of effective protection by ITNs at village and field (n = 141)

Of 141 survey participants, 82.2% reported having used an ITN the night before the survey. However, only 7.8% (9/116) said they actually slept under a hung-up ITN and the majority (92.2%, 107/116) reported to have used their net as a blanket (Table 1). Further analysis of ITN use (Fig. 1) revealed that only 28.4% (40/141) of participants were estimated to be protected by ITNs at village houses. The main reason was that people slept near the kitchen fire for warmth and believed in the mosquito repellent effect of smoke. After accounting for those who additionally slept at plot huts without having or hanging up their net, only 6.3% (9/141) of participants were estimated to be effectively protected by ITNs both at village houses and plot huts. Considering 12.1% (17/141) of participants who were protected at the village house and did not have a plot hut, a total of 18.4% (26/141) of all survey participants were estimated to be effectively protected by ITNs.

### Factors associated with ITN use

#### Working practices and multiple residences

M’nong households traditionally used swidden agriculture which involved clearing plots of forest land and rotating every five to six years. In 1991, the government legitimized its role in forest management through reinforcing a national law on forest protection; and in 2004 swidden agricultural practices and hunting among other forest exploitation activities were prohibited by local laws [35, 36]. Under these changes, from 2003 onwards, the provincial authorities established the commune of Tra Leng and its administrative area with identified geographic boundaries for sedentary agriculture (*e.g.* for cassava and hybrid rice) and settled Ca Dong and M’nong villages there. Swidden agriculture and subsistence activities were discouraged by the government, and hunting and logging were made illegal.

The survey showed that the majority of households (97.5%, 78/80) owned a field and that the average time to commute on foot between the village and the field was over one hour (Table 2). 71.2% of HHLs (57/80) reported having a plot hut at their field. Participants explained that households divided time between villages and fields, with children usually staying in the village to attend school, and adults staying at fields for farming. The median duration of stay at fields was 19 consecutive days, ranging from 2 to 56 days (Table 2). 54.3% of HHLs (31/57) who had plot huts in the field said that they did not bring any ITNs to their plot huts. A common reason was having an insufficient number of ITNs to cover for all household members in both locations. Some participants said they often left intact ITNs at their village house for children to use, and the broken ones, if any, would be kept at the plot hut. Additional reasons for not bringing ITNs to the fields included: (1) short duration of stay at fields that did not warrant bringing the nets; (2) insufficient space inside the plot hut to hang up a net; and (3) the reticence to get the nets dirty or torn. According to some participants, male adults often stayed overnight in the forest for logging and hunting, exposing themselves to malaria. Stays in the deep forest, usually in groups of men, lasted several days. These groups rarely brought ITNs to stay in the forest because of the hardship endured while moving around (a need to travel “light”), and the difficulty of finding a suitable spot to hang up a net or a hammock. Instead, hunters would bring canvas (plastic sheets) to use as both a shelter and a blanket.

**Table 2:** Forest activities, multiple residences and ITN use amongst households (N = 80)

	n	%	95% CI
Households who reported that they owned a field (cinnamon plantation)			
Yes	78	97.5	[94.0; 1.0]
No	2	2.5	[0.9; 5.9]
Households who reported that they owned a plot hut at the field			
Yes	57	71.2	[60.0; 80.8]
No	23	28.8	[19.1; 39.9]
Reported periods of stay at fields			
Prepare the field for a new crop	18	22.5	[13.9; 33.2]
Whole year	27	33.7	[23.5; 45.1]
Harvesting time	1	1.3	
Other (periodic removal of grass, visiting the field)	34	42.5	[31.5; 54.0]
Median number of traveling time (in minutes) between the village and the fields	(range); IQR 74 (10; 180); IQR: 40–120		
Median number of days staying at fields	(range); IQR 19 (2; 56); IQR: 10–28		
Households who reported that they brought ITNs to the field to sleep in (N = 57)			
Yes	23	40.3	[27.5; 54.1]
No	31	54.3	[40.6; 67.6]
Sometimes	2	3.5	
No answer	1	1.7	

#### Sleeping places

Sleeping around the kitchen fire was common practice in the area. Part of this practice was due to the cold and humid climate, and the house structure (see housing conditions). Participants also explained that sleeping around the kitchen fire was linked to their traditional sleeping arrangements and animistic beliefs.

“We are used to sleeping in the kitchen. We believe the kitchen is a sacred place where the spirits stay, so we sleep there to be close to them. In my family, my parents worshipped the spirits and dedicated the kitchen to them. I do the same, I follow what my parents did. In the kitchen, I placed three stones to make a cooking fire. A lady shaman helped me invite the spirits to live in the stones. Twice a year, I make a sacrifice to the spirits.” (IDI, farmer).

ITNs were rarely hung up in the kitchen/cooking space due to fire hazards and the lack of space to properly hang up a net. Indeed, partially burnt ITNs were sometimes observed. Up to 60.0% (48/80) of HHLs answered that their household members, predominantly elders, often slept around the kitchen fire (Table 1). The majority (75.0%, 60/80) of HHLs believed that kitchen smoke would repel small insects, including mosquitoes, therefore sleeping under a hung-up net in the kitchen was deemed unnecessary.

#### Housing conditions

In villages, the government provided households wooden planks to build houses directly on the ground instead of on stilts, as was the case for the Ca Dong and M’nong traditional long houses. Furniture, including beds, was  required to sleep inside the government-supported houses, but the cost was not affordable to everyone. At fields, plot huts were on stilts, made of wood, bamboo, and forest leaves, and often small. Extracted data from the cohort study showed that village houses were made of temporary materials including wooden walls (100%, 80/80) and tin roofs (93.8%, 75/80). The house structures were open with gaps between wooden planks constituting the floor, and between the walls and the roofs, allowing mosquitoes to easily enter and exit. Due to the open structure, houses were quite cold and humid at night, particularly in the rainy/malaria season. Participants explained that the lack of financial means limited their ability to buy sufficient blankets to keep warm at night. In the survey, 75.6% (59/78) of HHLs reported using their broken ITNs either as pillows (49.2%, 29/59) or blankets (33.9%, 20/59) (Table 1).

#### ITN lifespan

**Fig. 2:**
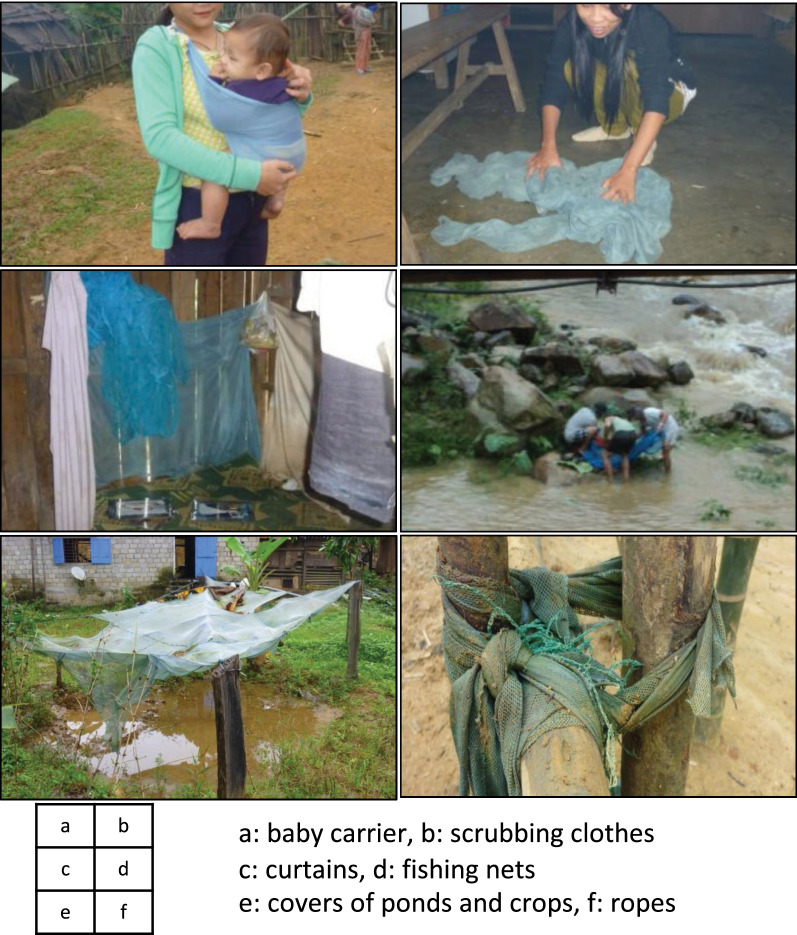
Alternative use of ITNs. (photos taken during fieldwork between April 2009 and January 2011). **a** baby carrier, **b** scrubbing clothes, **c** curtains, **d** fishing nets, **e** covers of ponds and crops, f: ropes

The qualitative strand identified several reasons for damaged ITNs after a short period of use. Participants explained that the netting was too hard, and easily broken when people removed the net from wooden walls or bamboo floors, or when children played inside the house. The open housing structure allowed animals, particularly chickens, to enter the house, and they also caused additional damages to the net. The house structure also exposed ITNs to soil and dirt, and together with children’s urine and faeces (households could rarely afford buying diapers), these factors contributed to more frequent net washing. 35.9% (28/78) of HHLs responded that they washed their nets every week (Table 1). Frequent washing and hanging bed nets outdoors to air-dry them with animals and children playing also caused additional damages and further shortened ITN lifespan. Damaged ITNs were not repaired or mended because people considered patches on the nets as a visible sign of poverty. Some participants said that damaged ITNs were not worth repairing because the hard netting caused skin irritation. In addition, they also preferred to use damaged nets for alternative purposes than sleeping (Fig. 2a–f).

#### Perceived causation of malaria

The local malaria control programme provided the population with health messages through printed materials and public campaigns focusing on the mosquito-malaria link and the benefits of sleeping under a net for malaria prevention. These messages in Kinh (Vietnamese) language were often not comprehended by local people. People linked fevers, including malaria fever, to an offense to the spirits or difficult living conditions at fields, such as intensive labour, poverty, poor housing and the lack of safe drinking water and a nutritious diet. The survey found that 43.3% (61/141) of participants identified that malaria was caused by mosquito bites. However, they answered that malaria could additionally be caused by the hot sun (52.5%, 74/141), hard work (48.9%, 69/141), and drinking bad water (41.1%, 58/141) (Table 3). Approximately one in five participants (19.1%, 27/141) answered that mosquito bites as the sole cause of malaria. Of participants who reported that they “always” or “sometimes” slept under a net, 60.3% (73/121) stated that they did so to protect themselves against malaria (Table 1).

**Table 3:** Knowledge of the cause of malaria (N = 141 individuals)

	n	%	95% CI
What is the main cause of malaria?			
Hard work	1	0.7	[0.1; 3.9]
Mosquito bites	61	43.3	[35.4; 51.5]
Spirits	1	0.7	[0.1; 3.9]
Do not know	76	53.9	[45.7; 61.9]
No answer	2	1.4	
Can too much hot sun give you malaria?			
Yes	74	52.5	[40.1; 56.9]
No	58	41.1	[43.1; 59.9]
Do not know	9	6.4	
Can heavy rain give you malaria?			
Yes	67	47.5	[43.4; 60.4]
No	62	44.0	[39.6; 56.5]
Do not know	12	8.5	
Can drinking bad water give you malaria?			
Yes	58	41.1	[36.3; 53.2]
No	72	51.1	[46.8; 63.7]
Do not know	11	7.8	
Can working too hard cause malaria?			
Yes	69	48.9	[44.2; 61.0]
No	62	44.0	[39.0; 55.8]
Do not know	10	7.1	

Table 4 summarizes the multi-variable analysis for appropriate ITN use, measured through self-reported ITN use (hung-up) the night before the survey. Significant potential factors identified by univariate analysis were gender, education, occupation, coverage of ITNs, coverage of blankets, knowledge of malaria causation, not sleeping near the kitchen fire, and not sleeping under a blanket the night before the survey. The multivariate adjusted analysis showed that four factors were independently associated with appropriate ITN use. More specifically, female participants were 8 times more likely than male (AOR = 8.08; p = 0.009) to report that “they always sleep under a hung-up net”; and so did participants who acknowledged the malaria causation (AOR = 7.43; p = 0.008). Participants who had sufficient coverage of ITNs (AOR = 21.69; p = 0.001) and those who reported that they did not sleep near the kitchen fire (AOR = 24.57; p = 0.001) were more likely to report appropriate use of ITNs. Despite large 95%CIs around the AORs due to the small sample size, the effects were found highly significant.

**Table 4:** Risk factor analysis for appropriate ITN use the night before survey (using survey logistic regression; N = 141)

Risk factors	n/N	%	95% CI	OR	95% CI	AOR	95% CI
Gender							
Male	51/67	76.1	[0.63; 0.85]	1			
Female	65/74	87.8	[0.76; 0.94]	2.26	[0.88; 5.80]	8.08	[1.73; 37.86]*
Education							
Illiterate	31/43	72.1	[0.55;0.84]	1			
Primary	41/49	83.7	[0.70; 0.91]	1.98	[0.75; 5.22]		
Secondary and higher	37/41	90.2	[0.76; 0.96]	3.58	[1.12; 11.48]*	–	
Occupation							
None (children, disabled)	15/23	65.2	[0.43; 0.81]	1			
Farmers	52/64	81.2	[0.70; 0.88]	2.31	[0.94; 5.62]		
Others (officer, business etc.)	42/46	91.3	[0.79; 0.88]	5.6	[1.78; 17.58]*	–	
Coverage of ITNs							
1–2.5 persons/net	90/99	90.9	[0.82; 0.95]	4.61	[1.60;13.31]*		
3–10 person/net	26/38	68.4	[0.49; 0.82]	1		–	
Knowledge of mosquito bites-malaria link							
No	88/112	78.6	[0.68; 0.85]	1		1	
Yes	26/27	96.4	[0.76; 0.99]	7.0	[0.90; 55.40]	7.43	[1.70; 32.41]*
Sufficient coverage of ITNs							
No	24/41	58.5	[0.41; 0.73]	1		1	
Yes	92/98	93.9	[0.87;0.97]	10.86	[3.84; 30.70]**	21.69	[4.61; 102.07]**
Sufficient coverage of blankets							
No	10/16	62.5	[0.35; 0.83]	1			
Yes	106/125	84.8	[0.75; 0.90]	3.34	[0.95; 11.79]	–	
Sleeping around the kitchen fire							
No	84/88	95.5	[0.88; 0.98]			1	
Yes	32/53	60.4	[0.43; 0.75]	13.781	[4.53; 41.91]**	24.57	[4.74; 127.22]**
Sleeping under a blanket the night before the survey							
No	12/19	63.2	[0.37; 0.82]	1			
Yes	104/122	85.3	[0.75; 0.91]	0.30	[0.09; 0.96]*	–	

*CI* Confidence interval, *OR* Odd ratio, *AOR* Adjusted odd ratio

*p < 0.1; **p < 0.001

## Discussion

This mixed-methods study shows the structural factors embedded in the local context and swidden agricultural setting that limited ITN use among the M’nong and Ca Dong communities. Despite relatively high ITN coverage and a high proportion of reported ITN use the night before the survey, after accounting for a combination of individual and household factors, *i.e.* the quantity of intact and available nets in the household, frequency and pattern of use (hung-up or as a blanket), sleeping practices (*e.g.* sleeping near the kitchen fire) and places (at village houses or fields), less than onefifth of the study population was estimated to be effectively protected by ITNs.

The study findings highlight the influence of social factors on ITN use, which has been reported by studies in a variety of contexts. A set of factors relates to the acceptability or appropriateness of specific bed net characteristics. For certain populations, the uptake of ITNs relates to product features such as smell, colour, texture, size, or how the design of nets meets user preference and expectations, *i.e.* privacy, ease of use, type of material considering climatic conditions, and net size in relation to housing size and structures [16, 37–44]. In Southeast Asia, the standardized design and features of LLINs were found challenging for uptake by indigenous populations who make up the majority of at-risk populations in the remaining endemic areas. In Cambodia, non-treated commercial nets were preferred over ITNs because the latter had a large mesh size allowing small insects to enter, and did not offer privacy for couples in shared sleeping spaces [33, 45]. In Myanmar, ITN use was limited by the hard material of ITNs, the limited choice in net size to accommodate variable family sizes and sleeping places, and the inadequate height of the net for people to sit comfortably [46]. In Vietnam, limited ITN use was linked to hard netting [41], the design of ITNs that was uncomfortable for users who slept on the floor (without a bed), and limited choices for net size for varied family and house size [47, 48]. These studies suggest a more appropriate ITN design and features that are better adapted to climatic conditions, variable family structures and sleeping places could increase the acceptance and appropriate use in minority populations.

However, beyond ITN characteristics, structural social factors directly affect net use. As shown in this study, poverty, mobility patterns, and multiple residences linked to swidden farming constrained effective ITN use and the product’s lifespan. In Southeast Asia, a unique factor among ethnic minority farmers is the multiple residency system whereby people live in more than one place, such as in the formal villages and combining  with homes at fields and/or rice paddies [15, 49]. In a similar ethnic minority setting to the M’nong, among the Ra-glai in Bac Ai district of Ninh Thuan province [15, 46], reasons for not using ITN were the limited indoor space at plot huts, poor air ventilation in government-subsidized houses and outdoor sleeping practices, including sleeping in the forest for (il)legal activities. In addition, the state’s socio-economic interventions such as repurposing traditional minority territory into government-managed forest land, have made the relationship between these minority groups and the state increasingly complex [50–52], representing another structural factor influencing the level of receptiveness of minority populations to state-led interventions, including those on disease control.

In sub-Saharan Africa, several structural factors limit ITN use, such as specific occupational nighttime activities like fishing, hunting, and brick-making [53, 54], sleeping outdoors for part of the night due to the heat [43], and frequent nighttime movements in and outdoors [55]. In the Peruvian Amazon, a qualitative study with the use of structured observation for sleeping spaces and bed net use highlighted multiple net entries and exits at night as a crucial factor for maintaining malaria transmission [56, 57].

In this study, the significant difference between the reported ITN use and the estimated effective protection of ITNs shows the importance of applying adequate research methods and measurements for social variability to inform public health measures. Standard indicators for ITN use offer the convenience of implementation and comparability across settings. The use of these indicators, *i.e.* responses to the question “did you sleep under a bed net last night”, across variable social contexts, however, leads to ignoring relevant heterogeneity and related contextual factors, the collection of “pseudo” data, and the construction of incomplete knowledge about the complexities of malaria elimination [58]. As illustrated in this study, the association between malaria control and increased forest control by the government poses an additional challenge for research on exposure, mobility, and activities in the forest. In such a minority setting, the use of qualitative and ethnographic research approaches to designing a quantitative survey is required to operationalize the concept of net protection specific to the local context, thus improving the validity of the instrument for quantitative data collection. Triangulation of qualitative and quantitative methods was applied in the study to assess the extent to which people are effectively protected from mosquito bites while sleeping at night. This measurement process is far from straightforward. The assumptions of effective bed net use are that people sleep (1) all night, (2) under a properly hung-up net, (3) that is still somewhat intact, and, do so (4) continuously across time and place. Empirical evidence suggests self-reported bed net use should be interpreted with caution [59, 60]. In the study, qualitative methods were employed to examine existing assumptions related to ITN use as well as to assess relevant variables for the operationalization of ITN use as a complex concept. In Cambodia and Vietnam, researchers reported a major difference between self-reported net use the night before the survey and observed net use at people’s homes [33, 61]. Other mixed methods studies in Southeast Asia, including qualitative methods before conducting a survey, resulted in the inclusion of a series of local factors to measure effective bed net use such as mobility between fields and villages, availability and use of intact bed nets at home and fields, and evening outdoor activities [21, 47, 62–64]. In Zanzibar, qualitative methods were used to explore night-time activities and sleeping patterns in one study while another study used a combination of survey research and structured observation to identify the variation in levels of net use [65, 66]. In Ghana, direct observation was also used to study factors contributing to residual malaria transmission including night-time activities, outdoor sleeping, and variability in evening activities [55].

The study found the most important risk factor for inappropriate ITN use was the practice of sleeping around the kitchen fire for both spiritual and practical reasons (*e.g.* the cold and humidity). This practice was in line with M’nong social organization (*i.e*. open-structured houses on stilts and matrilineal sleeping arrangements) and an adaptation to local ecological conditions (such as the climate and local ways of repelling small insects). The second most important risk factor was insufficient ITN coverage. Similar to this study, studies in Cambodia and Vietnam also reported the issue of low coverage of ITNs among ethnic minority populations whose mobility patterns and multiple residence systems were unaccounted for by the national ITN distribution plan [15, 49, 62]. Finally, women and participants who acknowledged the link between malaria and mosquito bites were more likely to adequately use ITNs as compared to men or people with less knowledge about malaria. It has previously been shown that women were generally less at risk of malaria than men in forested areas of Central Vietnam [17, 20]. In the setting of Tra Leng where people lived in open-structured housing with cold and humid weather, the knowledge of malaria causation alone was unlikely to influence the practice of sleeping near the fire. In addition, poor housing and living conditions influenced the way local people used and maintained their nets and consequently their expected protective effect.

**Fig. 3:**
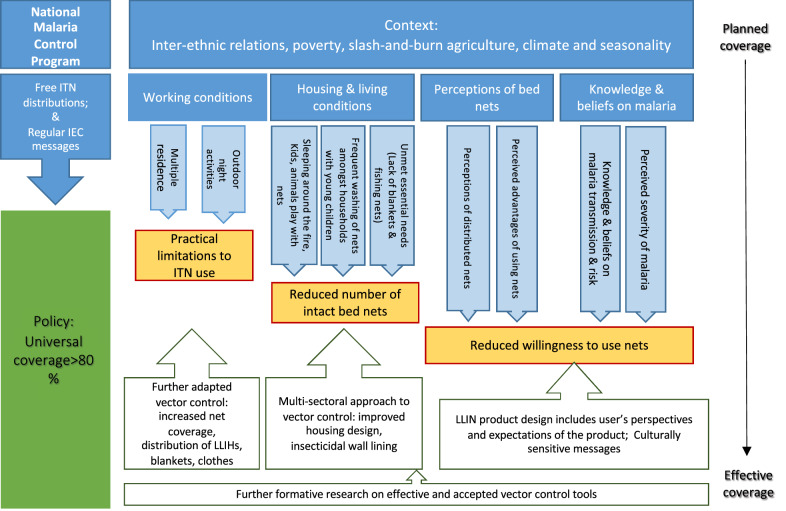
Intervention model to address contextual factors influencing ITN use

In Vietnam, the main challenge to malaria elimination is transmission in forested areas where many ethnic minority groups inhabit. Based on the study results, a theoretical model is proposed for effective bed net use and related recommendations which includes a formative research process to adapt vector control tools, improve the product design based on end-user perspectives, and include multi-sectoral approaches to vector control (Fig. 3). For the distribution of ITNs in ethnic minority populations, specificities such as type of nets (for single and double bed size) and the quantity per household, should be adjusted according to the local residence system, sleeping places, and arrangements of the local population. In similar settings to Tra Leng and Tra Cang communes, the NMCP could provide the population with additional tools such as insecticide-treated hammock nets [67] as well as, for example, insecticide-treated clothing [68, 69] and blankets [70]. To increase the uptake of these vector control tools, the product design and distribution should include an iterative process of dialogues with communities to test, improve, and finalize the product prototype before mass production and distribution. The introduction and distribution of additional/new tools should be supported by effective health communication strategies, including culturally sensitive health messages in local languages to avoid further perpetuating views of ethnic minorities as backward slash-and-burn farmers or disease carriers. There is no evidence of successful health IEC or behaviour change communication among ethnic minority populations in Vietnam. An additional factor that can lead to higher net use, as shown in a variety of settings, is designing bed nets or other vector control tools in such a way that they offer additional benefits besides malaria prevention such as increased privacy [33], improved sleep and protection from other insects [71], or in some settings with open housing, bed nets are required to take the function of inner walls [16]. In certain sub-Saharan African settings, improved housing has been shown as a complementary intervention to interrupt malaria transmission [72–75]. Existing evidence supports the hypothesis that improved housing interventions might be an additional solution for poor ethnic minority groups. However, to be effective in the Vietnamese context, these interventions will require the inclusion of social and cultural perspectives as well as the acceptance of the intervention by both the state and ethnic minority groups.

## Limitations

This study has a number of limitations, with the most visible one being the time between data collection and publication (> 10 years). Despite this, the study findings are still relevant for Vietnam. The NMCP has continued to include ITN distribution as an important element of vector control in the elimination strategies to 2030, and recent studies in ethnic minority settings have continued to report on limited uptake of bed nets [41, 47].

In terms of the survey response, outcomes of bed net use the previous night might have been inflated due to self-reporting. However, this inflation was likely to be homogenously distributed in the study population and therefore unlikely to have biased the estimates of the multivariate analysis. Factors limiting ITN use presented in this paper were specific to the minority populations of Tra Leng and Tra Cang communes, however, the operationalization of the concept of ITN use can be theoretically transferrable to similar ethnic minority settings in Central Vietnam. Indeed, the study population represents the challenges faced by several minority groups (*e.g.* Ra-glai) living in forested areas in Vietnam [15, 47, 61] and in the Greater Mekong Subregion [62, 76, 77], such as a forest-based livelihood, swidden farming, multiple residencies, mobility, the lack of nets, different local housing styles and conditions as well as high illiteracy and animistic beliefs, which are not addressed by the standardized interventions and approaches to malaria elimination.

## Conclusions

While knowledge of malaria and bed net coverage are important aspects of malaria prevention, it was mainly the hardship and poverty people endured that reduced ITN use, thus, exposing them to malaria. This study also illustrates how bed net use as a complex concept was operationalized and assessed in a minority setting. The study calls for the inclusion of an in-depth understanding of the local context to further improve the indicators for measuring ITN use.

## Data Availability

For the qualitative strand, the NVivo database with excerpts of the transcripts relevant to the study is available from the corresponding author on reasonable request. For the quantitative strand, the datasets used and/or analysed during the current study are available from the corresponding author on reasonable request.
